# Higher body mass index and lower intake of dairy products predict poor glycaemic control among Type 2 Diabetes patients in Malaysia

**DOI:** 10.1371/journal.pone.0172231

**Published:** 2017-02-24

**Authors:** Ping Soon Shu, Yoke Mun Chan, Soo Lee Huang

**Affiliations:** 1 Department of Nutrition and Dietetics, Faculty of Medicine and Health Sciences, Universiti Putra Malaysia, Selangor, Malaysia; 2 Malaysian Research Institute on Ageing, Universiti Putra Malaysia, Selangor, Malaysia; TNO, NETHERLANDS

## Abstract

This cross-sectional study was designed to determine factors contributing to glyceamic control in order to provide better understanding of diabetes management among Type 2 Diabetes patients. A pre-tested structured questionnaire was used to obtain information on socio-demographic and medical history. As a proxy measure for glycaemic control, glycosylated haemoglobin (HbA1c) was obtained as secondary data from the medical reports. Perceived self-care barrier on diabetes management, diet knowledge and skills, and diet quality were assessed using pretested instruments. With a response rate of 80.3%, 155 subjects were recruited for the study. Mean HbA1c level of the subjects was 9.02 ± 2.25% with more than 70% not able to achieve acceptable level in accordance to WHO recommendation. Diet quality of the subjects was unsatisfactory especially for vegetables, fruits, fish and legumes as well as from the milk and dairy products group. Higher body mass index (BMI), poorer medication compliance, lower diet knowledge and skill scores and lower intake of milk and dairy products contributed significantly on poor glycaemic control. In conclusion, while perceived self-care barriers and diet quality failed to predict HbA1c, good knowledge and skill ability, together with appropriate BMI and adequate intake of dairy products should be emphasized to optimize glycaemic control among type 2 diabetes patients.

## Introduction

Type 2 Diabetes (T2D) is one of the most common non-communicable diseases with growing incidence worldwide including Malaysia [1–4]. It is a well-established risk factor for cardiovascular diseases, with people with T2D having a higher cardiovascular morbidity and mortality [5]. The glycaemic control among T2D Malaysiane has been reported to be poor [6]. Achieving optimal glycaemic control requires a complex regimen of behaviours that must be followed consistently over a lifetime [7]. Insulin or medication administration and adjustment, self-monitoring of blood glucose (SMBG), and managing food intake represent significant behavioural demands [8]. Although the importance of lifestyle modification is highly emphasized to obtain optimal outcomes in diabetes, low compliance to lifestyle modification is frequently reported with a significant proportion of patients' diets remain poorly controlled [9–11]. Prospective studies have consistently shown poor adherence to the dietary recommendations for macronutrient intake, fruit and vegetable consumption in diabetic patients [12–14]. The persistent increasing trend in the total number of T2D patients warrants proper investigation into the determinants of poor glycaemic control among this population. Taking together this study aimed to identify whether perceived self-care barriers, diet knowledge and skills and diet quality contribute to HbA1c level among type 2 diabetics.

## Materials and methods

### Subjects and study location

This was a cross-sectional analytical study designed to identify factors associated with glycaemic control among individuals with T2D. All subjects were recruited from the Medical Specialist Out-Patient Department of Serdang Hospital, one of the tertiary hospitals in Malaysia. Inclusion criteriaincluded, aged 18–65 years old and had received diabetes care treatment for at least a year prior to the study. Patients who were suffering from severe illnesses such as end stage kidney disease and advanced stages of cancer, which may change/interfere with the nutritional behaviours; pregnant and lactating mothers; patients with frequent hypoglycaemic attacks (at least one attack per week) and modification in type or dosage of medication over the past three months were excluded from the study. Random sampling was used to recruit the eligible subjects into the study. Ethics approvals were obtained from the Research Ethics Committee of the National Medical Research Registry (NMRR) Malaysia and Universiti Putra Malaysia. The study subjects were given both oral and written explanation via subject information sheet and written informed consent was obtained from each subject before enrollment.

### Study instruments

All subjects were interviewed by the same researcher using a pre-tested structured questionnaire. Information obtained included socio-demographic background (sex, ethnicity, age, marital status, and household monthly income) while information on duration of diabetes, types of medication, presence of comorbid diseases as well as the latest glycosylated haemoglobin A1c (HbA1c) readings were obtained from individual medical reports as secondary data. Body weight of subject was measured using a TANITA electronic balance scale (TANITA-HD-302) to the nearest 0.1kg. The height of the subjects was measured by using a SECA body meter (SECA-206) to the nearest 0.1cm. Two measurements were taken for body weight and height and the average was recorded. Body Mass Index (BMI) was computed accordingly.

Diet knowledge and skill of subjects on their understanding on the importance of regular meal timing, skill on carbohydrate counting, and matching carbohydrate with physical activity were ascertained using a modified Personal Diabetes Questionnaire (PDQ) [15]. It is a clinically-focused and structured self-report measure that address the prescribed self-care regimen and current behaviour and future readiness to change [16–17]. Maximum possible score was 25 with higher score of diet knowledge and skill indicates better diet knowledge and skill. Perceived barriers to healthy eating, medication and self-monitoring blood glucose (SMBG) were assessed using a 24-item questionnaire, adopted and adapted from previous studies [18–19]. Examples of statements pertaining to diet, medication and SMBG barriers include, “Healthy foods are often not available when it is time for me to eat”, “I feel discouraged due to lack of results (e.g. no weight loss, high blood glucose)” and “feeling discouraged or dislike needles”, respectively. Participants were asked to rate from 1–5 (1 = 1 or more times per day; 2 = 4–6 times per week; 3 = 1–2 times per week; 4 = 1–3 times per month; 5 = never) the extent to how often a stated barrier has made it difficult for them to follow appropriate eating, medication or SMBG in the past 3 months. Higher summary scores indicate more perceived barriers, with a maximum cumulative score of 120.

Dietary intake of subjects was determined using a 122 structured listing of individual food items semi-quantitative food-frequency questionnaire. For each item on the food list, the subjects were asked to estimate how frequent the food was consumed for the past one month. Number serving for food groups of the subjects was determined based on national dietary guidelines (MDG 2010) [20]. The dietary quality of the subjects was measured using the modified Healthy Eating Index (HEI 2005) [21] which assesses conformance to national dietary guidelines (MDG 2010). The overall HEI score is the sum of 10 dietary components (grains & cereal products, vegetables, fruits, milk & dairy products, fish, meats & legumes, total fat, saturated fat, cholesterol, sodium, variety), weighted equally. Each component of the index has a maximum score of 10 (full compliance) and a minimum score of zero (lack of compliance). The score was calculated proportionately for in between responses. For all components, higher scores reflect better diet quality because the moderation components are scored such that lower intakes receive higher scores. The scores of the 10 components are summed to yield a total score, which has a maximum value of 100. The dietary quality of the subjects was classified into three categories namely, good (score > 80), need improvement (score 51–80) and poor (score < 50) [21].

### Statistical analysis

Analyses were performed using the IBM Windows Version 22 (Chicago, IL). Explanatory Data Analysis was carried out to determine the normality and homogeneity of the data. Unless otherwise specified, continuous variables are presented as mean ± standard deviation while categorical variables are expressed as percentage for each item. Multivariate analysis was performed to identify factors that predict the glycaemic control, with HbA1c as the dependent variables. The level of probability, p<0.05 was used to show the level of significance for all the tests.

## Results

A total of 155 T2D patients were recruited with a response rate of 80.3%. Respondents were made up of 46.5% males and 53.5% females (Table 1). The mean age of the subjects was 53.0 ± 9.4 years with the males having slightly higher (51.7 ± 9.9 years) mean age than the females (54.0 ± 8.9 years). Approximately one-third of the respondents were older adults. Majority of the subjects (86.5%) were married, with a comparable number of married females and males. Almost all subjects had attended at least some formal education at the primary school level but 2.6% had not received any formal education. The mean number of years of education was 10.5 ± 4.2 years with males having slightly more years than females (t = 5.217, p = 0.001). There were higher numbers of male subjects (44.4%) who had attended tertiary school compared to female subjects (15.7%). The mean duration of diabetes diagnosis was 10.4 ± 10.7 years. Most of the subjects (45.8% males and 38.6% females) had been diagnosed as diabetics for at least 1 to 5 years. A majority of the subjects have more than one comorbid diseases, with only 10.0% without any comorbid disease. The overall average medication compliance rate of the subjects was 94.1 ± 13.9% with majority of the subjects (72.3%) being compliant with the prescribed medication. There were 81.8%, 69.7% and 52.2% of subjects who complied with oral medication, oral medication plus insulin and insulin injection respectively.

**Table 1:** Distribution of subjects according to selected characteristics (n = 155).

Characteristics	Male (n = 72)	Female (n = 83)	Total (n = 155)
**Socio-demographic Factors**			
**Age (Years)**	51.7 ± 9.9	54.0 ± 8.9	53.0 ± 9.4
**Marital Status**			
Single	5 (6.9)	2 (2.4)	7 (4.5)
Married	65 (90.3)	69 (83.1)	134 (86.5)
Divorced	1 (1.4)	0 (0.0)	1 (0.6)
Widow/ widower	1 (1.4)	12 (14.5)	13 (8.4)
**Education (years)**	12.3 ± 3.6	9.0 ± 4.1	10.5 ± 4.2*
None	0 (0.0)	4 (4.8)	4 (2.6)
Primary	7 (9.8)	24 (28.9)	31(20.0)
Secondary	33 (45.8)	42 (50.6)	75 (48.4)
Tertiary	32 (44.4)	13 (15.7)	45 (29.0)
**Body Mass Index (BMI) (kg/m2)**	29.70 ± 5.96	29.09 ± 6.39	29.38 ± 6.18
**Total HEI Score**	69.11 ±11.00	94.1 ± 14.9	71.53 ±10.16*
**Diet knowledge and skill score**	9.63 ± 3.96	9.84 ± 4.21	9.74 ± 4.08
**Perceived self-care barrier score**	105.54 ±12.98	107.70 ± 9.40	106.69 ± 11.22
**HbA1c (%)**	9.12 ± 2.24	8.94 ± 2.26	9.02 ± 2.25
**Medical related Information**			
**Duration of diagnosis (years)**	9.5 ± 8.0	11.2 ± 12.6	10.4 ± 10.7
**Presence of comorbid diseases**			
None	8 (11.1)	7 (8.4)	15 (9.7)
Hypertension	12 (16.7)	14 (16.9)	26 (16.9)
Hyperlipidemia	10 (13.9)	14 (16.9)	24 (15.5)
Multiple comorbid diseases	40 (55.5)	48 (57.8)	88 (56.6)

Data presented as mean ± SD or frequency (percentage)

* significant different between sex at p<0.01

Mean BMI of the subjects was 29.38 kg/m^2^ with majority (60.0%) of the diabetic subjects being obese, 24.5% overweight and only 14.2% had normal BMI. Besides, mean HbA1c was 9.02 ± 2.25% indicating that subjects were at high risk of diabetes complications. Only 28.4% of the subjects were able to achieve normal target of HbA1c below 7.5%. Almost a third (32.9%) of the subjects had high levels of HbA1c while 38.7% had HbA1c > 9.5% which predisposed them to very high risk of diabetes complications.

**Fig 1:**
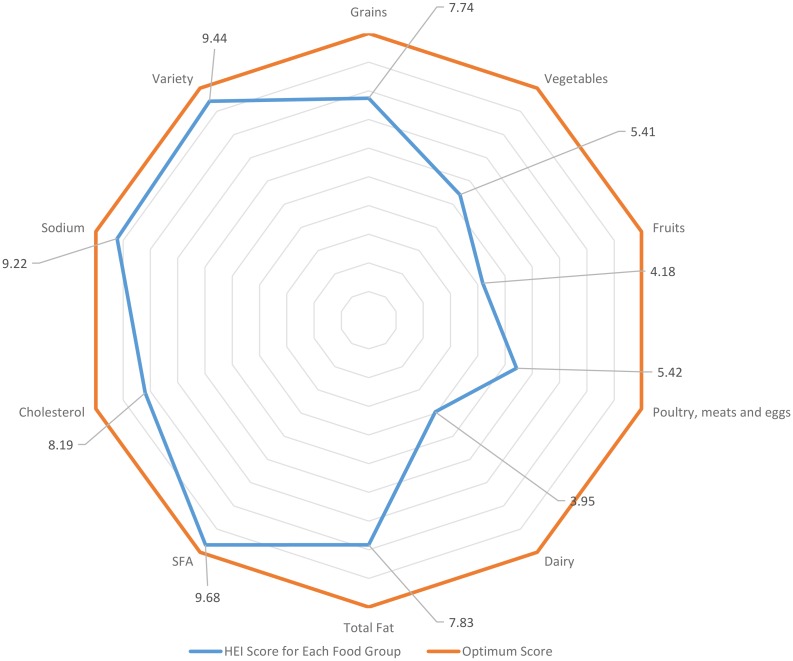
HEI of subjects according to food groups.

Despite a mean total HEI score of 71.53 ± 10.16, the overall diet quality of the subjects was unsatisfactory, with a majority of them (76.8%) needing to improve their diet quality and only 1.9% had good diet quality. As shown in Fig 1, adequacy of intakes in several food groups namely fruits, milk and dairy products and vegetables were relatively low compared to other food groups. Given that a score of 10 is optimum intake, mean HEI scores on vegetable, fruit, meat, poultry, fish, and legumes as well as milk and dairy products were only 5.41, 4.18, 5.42 and 3.95, respectively. The mean diet knowledge and skill score of the subjects was a low of 9.74 ± 4.08. There were 26.5%, 36.8%, 24.5% of the subjects whose dietary knowledge and skill were very low, low or medium level, respectively. Assessment on perceived self-care barrier on the other hand showed that majority of the subjects (93.5%) have the barrier scores in the range of 91–120, indicating that subjects had severe constraints to good self-care.

As presented in Table 2, the predictors for poor HbA1c were BMI, medication compliance, diet knowledge and skill scores, poor intake of milk and dairy products and increased intake of vegetables which in total contributed significantly to HbA1c level with the final model explaining 20.0% of the variables (R = 0.517, Adjusted R^2^ = 0.200, F = 3.953), with the estimated model: HbA1c = 12.41 + 0.063 BMI—0.026 medication compliance—0.125 diet knowledge and skill score—0.601 milk and dairy products intake + 0.286 vegetables intake. The remaining of 80.0% of the variance may be caused by factors such as physical activity, professional factors and others which were not studied in this study. Other factors such as self-care barriers or total HEI score (measures for diet quality) failed to predict HbA1c significantly.

**Table 2:** Multiple linear regression for factors contributing to HbA1c.

Predicted of Risk Factors	Unstandardized Coefficients		T	Sig.
	B	Beta		
Constant	12.41		5.408	0.000
BMI	0.063	0.172	2.234	0.027
Medication compliance	-0.026	-0.226	-2.102	0.037
Diet knowledge and skill score	-0.125	-0.163	-2.996	0.003
Milk and dairy products intake	-0.601	-0.205	-2.680	0.008
Vegetables intake	0.286	0.197	2.453	0.015

R = 0.517, R^2^ = 0.267, R^2^ adjusted = 0.200, F = 3.953, Sig-F = 0.000

## Discussion

The mean age of the subjects in the current study was slightly lower than other local studies among diabetics [6, 22–24]. In the current study, subjects were knowledgeable and were able to seek appropriate treatment and management for their diabetes as majority of them had some formal education. The mean duration of the diabetes diagnosis was in line with other local studies [25]. The increasing duration of the diagnosis of diabetes is an alarming sign for proper investigation and diabetes management to prevent higher rates of diabetes-related complications and premature mortality. By using the non-compliance definition from other studies [26–27], the current study reported almost one third of the subjects failed to comply with the medication prescribed. This finding was in agreement with several studies which found that medication compliance ranged between 36–93% [28–29]. Majority of subjects were able to discipline themselves to comply with oral medication but not insulin. Approximately half of the subjects failed to comply with the daily insulin injections. With the increasing complexity of the medication, a declining trend of compliance has been observed in the current study. The possible reasons attributed to non-compliance in both oral medication plus insulin and insulin only groups were the fact that the subjects had difficulty in assessing accurately insulin levels or insulin injection [30–32] and subjects’ own perception or emotional phobia towards insulin [33–35]. Non-compliance to drug prescription is highly prevalent and had been linked to increase in morbidity, mortality, and medical treatment costs. Thus, strict supervision and proper guidance is highly needed to improve low compliance to insulin injection.

Majority of the subjects were either overweight or obese regardless of sex. Similar scenario was reported in other countries [36–37]. Given that obesity reduces insulin sensitivity [38] and predisposes individuals to abnormal cardio metabolic profiles such as increased waist circumference with increased visceral adiposity and inflammatory adipokines, insulin resistance, elevated triglycerides, decreased high-density lipoprotein cholesterol, and lead to hypertension [39], there is an urgent need for weight management intervention. Mean BMI of subjects was higher than that of the National Diabetes Registry Malaysia (2009) [40]. Such finding definitely warrants further investigation.

Studies had consistently reported low vegetable intakes among Malaysian adults [4, 41–42]. The current findings are in concordance with Federal Agriculture Marketing Authority of Malaysia who reported the reduction in the consumption of vegetables per capita for Malaysians from 2006 to 2013 [43]. The reason for the decline in the consumption of vegetables may be due to bitterness, pungency and astringency compounds in vegetables [44–45]. The HEI scoring for fruit group was low (4.18 out of 10), as 81.9% of the subjects were unable to achieve two servings of fruits per day. These findings however is on contrast to reported increase in per capita fruits consumption over time for Malaysian [43]. However, such findings were not unexpected as earlier study documented that diabetes patients will try to refrain from having fruits in their meal as fruits are considered “too sweet” for them [46]. Despite consumption of milk per capital for Malaysian was relatively higher than Thailand, China and the Republic of Korea [47–48], inadequate intakes of milk and dairy products was evident in the present study as a majority of subjects (83.9%) failed to fulfil their minimum daily recommendation. Our finding was in congruent with the national dietary survey where only 15% of the Malaysian consumed milk on daily basis [41]. Milk and dairy products are not habitual Malaysian food items unlike Western populations. The belief that dairy products are fattening [49] and hence can impaired good glycaemic control among diabetics can partly explain the low consumption of milk and dairy products among diabetics in this study. Earlier local studies also documented that dairy products were the least-frequently consumed foods among T2D patients [46]. With regards to the protein group (meat, fish, poultry, eggs, legumes), a subgroup analysis showed that 76.1% of the subjects had adequate intakes of poultry, meats and egg. This is in-line with national surveillance studies that documented high prevalence of poultry and eggs product consumption among the adult population [41]. Consumption of fish and legumes however were inadequate where a total of 41.3% and 48.4% of the subjects failed to achieve recommended servings for fish and legumes, respectively (data not shown). The overall diet quality of the subjects was unsatisfactory especially in the consumption of vegetables, fruits, fish and legumes as well as milk and dairy products. Therefore in this study many subjects at the “need improvement” level of diet quality. Proper dietary guidance is needed to improve the subjects’ diet quality, to reduce their glycaemic levels [50–51].

Diet knowledge and skill level of the subjects in the present study was lower than other studies [52–53]. Subjects had inadequate diet knowledge and skill in determining appropriate food choices based on nutrients content. On the other hand, given that continuous motivation intervention is crucial for diabetics to choose healthy diets with actual food portion size, using foods exchange list and food calories in order to achieve better glycaemic outcome [54–55], health care providers especially dieticians should emphasize counselling approach which could help to increase diet knowledge and skill among diabetics.

The present study reveals high prevalence of study subjects who had “very high” perceived self-care barrier scores. The barrier score was relatively higher compared to the several studies [56–58]. This could be related to subjects’ depression, low socio-economic status, and lack of diabetes related knowledge. Long-term exposure to the disease can lead to the development of hopelessness, discouraged and disappointed if SMBG always gave an undesirable readings and uncontrolled blood glucose levels as reported by Lin *et al*. [59] and Ong, Chua and Ng [60]. Better self-care outcome mainly depends on the patient’s knowledge of self-care which also include knowledge of the disease, health-related and care-seeking behaviour [61–63] which are guided and determined by individually and culturally defined beliefs about health, illness and health-care [64].

Mean HbA1c of subjects was higher than other local studies [6, 65–66] putting them at high risk of diabetes complications. A high proportion of the subjects had HbA1c > 9.5% reflecting poor control of glycaemic level according to World Health Organization [67]. Only approximately 10% of the subjects achieved HbA1c of less than 6.5% and this percentage is relatively lower than other local studies [6, 65–66]. Given the overall high mean level of HbA1c and proportion of subjects with poor controlled glycaemic level as well as the low 10% of subjects achieving targeted HbA1c range, it is worth noting that the glycaemic control among the subjects is poor, despite the good compliance rate for medication. This highlights the urgency for the health care providers to review the treatment approach (e.g. dosage of medication, interval of follow-up, multi-disciplinary approach) for the diabetics.

It is interesting to note that higher intake of milk and dairy products, diet knowledge and skill score and compliance to medication while lower BMI and vegetable intake were associated with lower HbA1c, hence better glycaemic control. Higher medication compliance rate may reduce or control the glycaemic level via several different biological mechanisms. Several research supported the finding of the present study that good adherence to medication were strongly associated with lower HbA1c levels [68–71]. Diet and knowledge score was inversely significantly correlated with HbA1c level. Individuals with better dietary knowledge and skill enabled them to follow the dietary recommendations and were more likely to make the correct food choices and refusing foods high in sugar, calories, and fat [53]. The findings of this study have implications for patient education and clinical practice in Malaysia. By providing the required resources, glycaemic outcome is expected to improve. Body Mass Index was positively associated with higher reading of HbA1c. This emphasizes the importance of weight management in glycaemic control as majority of the subjects were overweight or obese.

While total diet quality did not contribute to HbA1c significantly, our findings was in congruence with a majority of the previous prospective studies [72–74], systematic review [75] or meta-analysis [76] which reported an inverse relationship between intakes of dairy product and risk of diabetes, but not in others [77–80]. There is an increasing interest in the potential role that dairy products play in diabetes etiology. Previous studies showed that milk proteins have insulinotropic properties and appear to induce rapid release of insulinotropic amino acids and incretin hormones [81–82], which may explain its benefit to glycaemic control. The discrepancies in findings with others [77–80] could be largely be attributed to the used of different types of dairy products in the different studies, with evidence generally favoring the low fat dairy and low fat fermented dairy product specifically yogurt, compared to whole milk [73, 83–85]. Fat content especially saturated fat in dairy products is generally being thought of as being able to offset the benefits of the potentially protective dairy components such as calcium, magnesium, vitamin D and whey proteins [75]. Nonetheless the present study did not study the influence of the types of dairy products on glycaemic control, because evidence favors the consumption of low fat dairy products compared to whole milk dairy products [79, 84–85]. Malaysian are in general non-habitual milk drinkers with milk consumption falling markedly among the children and adolescents. The present findings add to the evidence that low fat dairy products fit well into a healthy eating pattern and hence consumption should be promoted. As dairy products are diverse in structure, composition, and usage and are produced by a variety of methods, including fermentation, further research is warranted on the specific type of dairy products in relation to glycaemic control to elucidate its potential clinical role on optimal glycaemic control among diabetes patients. On the other hand, as there are studies showing that dairy fats may be more beneficial than other animal-derived fats in modulating risk of diabetes [86–87], suggesting food sources of fat instead of type of fats may be more imperative in determining risk of diabetes. Hence, more research that spans nutritional epidemiology and dietary public health that underpins the possible associations between dairy products consumption and glycaemic control are deemed necessary.

Intake of sufficient amounts of fruit and vegetables is recommended as a part of a healthy diet, in view of the presence of considerable protective constituents, including potassium, folate, vitamins, fiber, anti-oxidant content and phenolic compounds [88–89]. However, the mechanisms by which fruit and vegetables reduce the risk of type 2 diabetes have not been precisely elucidated. To date, many epidemiological studies have examined the association between fruit and vegetable intake with risk of diabetes or glycaemic control and the results are not entirely consistent. While some studies showed an inverse association with risk of diabetes or lower HbA1c with higher intakes of total fruit [90–92] and total vegetables [93–94], other studies did not [78, 95]. Our data suggest that fruit consumption is not associated with better glycaemic control in this population. Other studies have found similar results [94, 96–99]. The high fructose content of fruit may counteract the protective effect of antioxidants, fiber, and other antidiabetic compounds of fruit [98] as sugars containing fructose have been suggested to play a major role in the development of hypertension, obesity, diabetes and metabolic syndrome [100]. More research is needed to investigate the association between fructose content in fruit and health outcomes. On the other hand, our findings of a positive contribution of vegetables intake towards higher HbA1c was not seen in other studies [90, 93, 99, 101] and should be interpreted cautiously. We do not have a ready explanation for this finding but are speculating that the common cooking methods for vegetables in this population may partially explain this unexpected finding. Having vegetables consumed in a salad is not common in Malaysia as vegetables are often stir-fried or cooked with water or coconut milk. The high total fat or saturated fat content of cooked-vegetables may offset the benefits of other components of vegetables such as lignans, phytates and polyphenols, which are known for their antioxidant properties. The present study reveals that it is insufficient to convey public health messages about overall vegetable intake, but that a more direct messages identifying the type of vegetables and how they should be prepared are required. We suggest that caution should be observed in the recommendation of vegetable intake in an effort to provide healthier options. Besides vegetable intakes, dietitians and nutritionists should highlight the importance of preparation of vegetables in favor of raw, less oily, and coconut milk free cooking methods. On the other hand, as only total vegetable intakes was assessed in this study without information on the consumption of green leafy vegetables, the actual correlation between vegetables intake and glycaemic control cannot be elucidated clearly. Protective role of vegetables on risk of diabetes depends on type of vegetables. While green vegetable have been found to reduce risk to diabetes [90–91, 95], total vegetable intakes, yellow and red vegetables were not found to be beneficial in several studies [90–91]. In this study there was no significant correlation between HbA1c with age, education, income, duration of diabetes diagnosis and self-care barrier score in the multivariate analysis. These findings failed to support earlier studies [102–106] where number years of education, income, duration of diabetes diagnosis or self-care barrier were correlated with glycaemic control.

This study was in agreement with Jansen *et al*. [107] who reported that variance of HbA1c predicted by patients’ characteristics was relatively low compared to genetics factors. On the other hand, despite the variance of HbA1c predicted in this current study being lower than that reported by Egan *et al*. [108], it was comparable with other studies which reported that the variance of HbA1c predicted only ranged from 12–22% [109–111]. The dissimilarities of the findings may be attributed by difference in the type of predictors included in the model. The variance in the contribution of HbA1c in the present study can be further improved with large-scale populations recruited.

## Conclusion

Despite self-care barriers and diet quality failed to predict HbA1c, higher diet knowledge and skill score significantly contributed to better glycaemic control. On the other hand, factors that were found to predict poor glyceamic control are highly modifiable (BMI, medication compliance, diet knowledge and skill score, intakes of milk and dairy products). This study provides an insight on glyceamic control among diabetic patients, which can be a good reference for future studies. It is believed that through appropriate strategies, the glyceamic control among the type 2 diabetics can be greatly improved.

## Limitation of the study

This was a cross-sectional analytical study and the cause and effect relationship cannot be determined. Another limitation of this study was the use of non-probability sampling method in the selection of study location. It may not be a representative study that could be generalized for the whole population of T2D patients. Besides, this study only involved a small-scale population which could be too small to represent the whole population of the type 2 diabetes mellitus patients. Dietary recall bias where subjects could not remember clearly the frequency or type of foods that they have consumed in the past one month could have been a shortcoming in this study. However steps like the use of a validated food frequency questionnaire was used to overcome some of the limitations.

## Supporting information

S1 Dataset Data sps. (XLSX)
